# The Brain of the Fossil Man of Chapelle-Aux-Saints, Correze

**Published:** 1911-10

**Authors:** 


### The Brain of the Fossil Man of Chapelle-aux-Saints, Correze

Boule and Anthony, *L'Anthropologie,* Volume 22, No. 2, have studied the fissures, sulci and cerebral and cerebellar development by the device of making a cast from the interior of the skull. Like the Neandeithal and Spy fossils, the brain is long, wide and flat, differing widely from the majority of present human brains, though resembling certain microcephalic types and being intermediate between the ordinary present or even prehistoric brain and that of the anthropoid apes. The groove for the superior longitudinal sinus is relatively slightly developed, indicating that man has developed the cerebrum partly by encroachment upon this space.

The fissure of Sylvius is broadened anteriorly, apparently indicating a certain degree of extrusion of the Insula, recalling a form existing in man in a certain stage of ontogenic development. The fissure of Rolando presents characteristics also intermediate between man and the anthropoids.

Following the method of Wagner and Vogt, of estimating the dimensions of the various lobes from their endocranial surfaces, the same conclusion is reached. There is a beak, projecting from the anterior portion of the cerebrum, into the ethmoid fossa—olfactory lobe? The general conclusions drawn are that the man of Chapelle-aux-Saints had a brain well developed for ordinary sensation and motion, but that the frontal, intellectual centers were rudimentary. From the considerably greater projection corresponding to the left parieto-temporal region and the angular gyrus, he was probably strongly right-handed. As to speech, the authors are non-commital though holding on general grounds that this faculty was rudimentary.

Note. As there has been much discussion as to whether man was originally ambidexterous, as to the time when right-handedness developed, and whether this trait is to be regarded as progressive by specialisation of function, or retrogressive, the observation in this particular is especially interesting, although it is unsafe to draw conclusions from a single specimen. It has been held that, as Hebrew and certain other ancient languages were written from right to left, instead of by the natural sweep of the hand away from the center toward the right, as in the cases of more modern languages, man was originally left-handed. However, most ancient languages were written indifferently, from right to left or vice versa, or in one direction for one line, turning in the opposite way for the next; in other words, starting each line below where the preceding one left off. This fact has, of course, been made an argument for early ambidexterity.

Such arguments are, however, on a par with the logic of the princess who, being told that, on account of the exigencies of war, the supply of bread had given out, replied "Then let us have toast." They presuppose a desk and some form of paper and fine writing implement. Early writing undoubtedly began with marking with a stick on sand or smooth dirt, or with a pebble on a flat rock. In tracing large characters in this way,—and it is hardly necessary to explain that they were rude pictures and arbitrary signs, not letters—it requires locomotion of the whole body to follow a line, not a mere sweep of the hand across a page. As one would naturally move forward, not backward, right to left writing would be the natural original method of a right-handed people.

